# Asymmetric *Wolbachia* Segregation during Early *Brugia malayi* Embryogenesis Determines Its Distribution in Adult Host Tissues

**DOI:** 10.1371/journal.pntd.0000758

**Published:** 2010-07-27

**Authors:** Frédéric Landmann, Jeremy M. Foster, Barton Slatko, William Sullivan

**Affiliations:** 1 Department of Molecular, Cell and Developmental Biology, University of California Santa Cruz, Santa Cruz, California, United States of America; 2 New England Biolabs, Inc., Ipswich, Massachusetts, United States of America; The University of Queensland, Australia

## Abstract

*Wolbachia* are required for filarial nematode survival and fertility and contribute to the immune responses associated with human filarial diseases. Here we developed whole-mount immunofluorescence techniques to characterize *Wolbachia* somatic and germline transmission patterns and tissue distribution in *Brugia malayi*, a nematode responsible for lymphatic filariasis. In the initial embryonic divisions, *Wolbachia* segregate asymmetrically such that they occupy only a small subset of cells in the developing embryo, facilitating their concentration in the adult hypodermal chords and female germline. *Wolbachia* are not found in male reproductive tissues and the absence of *Wolbachia* from embryonic germline precursors in half of the embryos indicates *Wolbachia* loss from the male germline may occur in early embryogenesis. *Wolbachia* rely on fusion of hypodermal cells to populate adult chords. Finally, we detect *Wolbachia* in the secretory canal lumen suggesting living worms may release bacteria and/or their products into their host.

## Author Summary

Filarial diseases affect over 150 million people in tropical countries. They are caused by parasitic nematodes like *Brugia malayi* that rely on their endosymbiont *Wolbachia* for their survival and fertility. These bacteria are a recognized drug target in the search for treatments killing adult worms. To understand the transmission of *Wolbachia* from the embryonic to adult stages, we developed new techniques to track these bacteria at the cellular and tissue levels. These techniques include immunofluorescence in whole mount adult tissues and embryos. We found that *Wolbachia* segregate asymetrically in specific cells, in a lineage-specific manner during early *Brugia* embryogenesis, and rely on cell fusion to subsequently populate the adult hypodermal chords. From the chords, the *Wolbachia* can be secreted in the secretory-excretory canal, suggesting that in addition to dead worms releasing the bacteria in the human body, living worms may also secrete *Wolbachia*, whose role in stimulating the immune system in filarial pathologies is now well established.

## Introduction

Filarial nematodes are the causative agents of human filariasis, affecting over 150 million individuals. The most pathogenic diseases, lymphatic filariasis and onchocerciasis, (river blindness) comprise a major cause of global morbidity in the tropics, with over 1 billion people at risk of these arthropod-transmitted infections [1], [2]. Three filarial nematode species are responsible for lymphatic filariasis: *Wuchereria bancrofti*, *Brugia malayi* and *Brugia timori*, causing pathologies that include hydrocoele and lymphoedema (elephantiasis). Onchocerciasis is caused by *Onchocerca volvulus*, leading to skin disease, “onchocercoma nodules” and visual impairment, including blindness. These parasitic nematodes rely on alpha-proteobacterial *Wolbachia* endosymbionts for development, viability and fertility (for reviews see, [3], [4]). This obligate dependence was first discovered using anti-*Rickettsial* tetracycline antibiotics, in *in vitro* and *in vivo* model systems. Treatments deplete *Wolbachia*, resulting in embryonic arrest and a decrease in microfilarial (larval) production [4]. Human trials with doxycycline or rifampicin provide evidence for long-term sterilization and macrofilaricidal (adulticidal) effects against both lymphatic filariasis and onchocerciasis [5]–[8].

*Wolbachia* play a significant role in the pathogenesis of filarial disease [9]–[14]. *Wolbachia* activate inflammatory immune responses, including antibody responses and induction of corneal keratitis in the case of *O. volvulus* infection, and are implicated in the inflammation response leading to blindness, induced by release of *Wolbachia* antigens from degenerating microfilariae [3]. In lymphatic filariasis, the major pathologies are attributable to death and destruction of adult worms within the lymphatic vessels and activation of innate inflammation; effects which are lost following antibiotic depletion of bacteria and absent from soluble extracts derived from filarial species naturally lacking *Wolbachia* such as *Acanthocheilonema viteae* [4].

To better understand the endosymbiont interaction with the parasitic nematode, it is of primary importance to characterize *Wolbachia* localization at the host tissue and cellular levels. The histology of the parasitic nematode *B. malayi* was established more than half a century ago by differential contrast microscopy (DIC) on whole mount adult specimens [15]. Subsequent DIC and electron microscopy studies carried out on cross sections revealed the presence of intracellular bacteria [16]–[20] which were only “re-discovered” and identified as *Wolbachia* decades later by phylogenetic analysis and genomic studies [21], [22]. *Wolbachia* are present primarily in the lateral hypodermal chords of both adult males and females and in the ovaries, oocytes and embryonic stages within the uteri of females. The absence of *Wolbachia* in the male reproductive system indicates that the bacterium is vertically transmitted through the cytoplasm of the egg and not through the sperm [19], [23].

Although *Wolbachia* are observed in all stages of the host life-cycle, there are significant variations in bacterial growth kinetics in host development [24], . Bacterial numbers remain constant in microfilariae (mf) and the mosquito-borne larval stages (L2 and L3), but the *Wolbachia* multiply rapidly beginning within the first week of infection of the mammalian host.

Features of the symbiotic relationship left unresolved include the localization and segregation patterns of *Wolbachia* during embryogenesis, which are essential to understanding the specific localization in adult somatic tissue and the germline. To address this issue, we developed fixation, immunofluorescent staining and imaging protocols to characterize *Wolbachia* in whole-mount *B. malayi* embryos and adult specimens at the tissue, cellular and sub-cellular levels. These studies demonstrate that *Wolbachia* localize to the posterior of the egg upon fertilization and segregate asymmetrically during early embryogenesis, in a lineage-specific manner, resulting in only a small fraction of the cells in the developing embryo containing the endobacteria. Specifically, *Wolbachia* concentrate in the C blastomere hypodermal descendants, and in the P blastomere germline precursors. The asymmetric and lineage-specific segregation of *Wolbachia* during the initial stages of embryogenesis resembles that of some *Caenorhabditis elegans* polarity and lineage-specific determinants, and suggests that *Wolbachia* may interact with the counterparts of these determinants in *B. malayi*.

This transmission pattern readily explains the tissue specific pattern of *Wolbachia* localization in the adult hypodermal lateral chords and female germline. The absence of the bacteria from the embryonic germ line precursors in nearly half of the embryos suggest *Wolbachia* loss from the male germline may occur during embryogenesis. We find that *Wolbachia* rely on fusion of hypodermal cells to populate young adult chords. We also detected *Wolbachia* in the lumen of the secretory-excretory canals embedded in the hypodermal lateral chords, suggesting that in addition to dead or degenerating parasites, live adult worms may also release bacteria and/or their products through this route into the host tissues.

## Methods

### Specimens

Living *B. malayi* adult male and female worms were supplied by TRS Laboratories (Athens Georgia). The worms were raised in jirds and the procedures described below were performed approximately 1 to 3 days after their removal.

### Adult Worm Fixation and Immunofluorescence

To prepare worms for whole mount immuno-fluorescence analysis, they were soaked in M9 buffer (see Buffers in Supplementary Experimental Procedures) for 30 seconds to allow them to uncoil and immediately placed in liquid nitrogen. M9 was then removed and replaced with (PBS+ Paraformaldehyde (PFA) 4% final- (Electron Microscopy Sciences)) 1/3+2/3 Heptane on a rotator for 30 minutes at room temperature. If required, worms were cut or open with a blade to expose the different tissues to antibodies prior to fixation. All fixation and immunostaining steps gave better results in eppendorf tubes in rotation compared to whole mount animals on slides.

For propidium iodide (PI) (Molecular Probes) DNA staining, worms were incubated overnight at room temperature in PBS + RNAse A (15 mg/mL, Sigma), in rotating tubes followed by PI incubation (1.0 mg/mL solution) for 20 minutes in PBS (1∶50) and a 5 minute wash. For DAPI DNA staining alone, fixed worms were simply pulled out of the tube with a curved needle, placed on a glass slide with thin needles in a line of PBS. The PBS was then aspirated and worms mounted into Vectashield with DAPI (Vector Laboratories) and left at 4 degrees overnight: DAPI penetrates all tissues and stains *Wolbachia* very well.

### Embryo Fixation and Immunofluorescence

To prepare embryos for whole-mount immuno-fluorescent analysis, females were cut into sections with a blade on a glass slide. The sections were collected and the slide rinsed with PBS and collected in an 0.5mL eppendorf tube. PFA and heptane were added as described above. The tube was vortexed for one minute at this step. After fixation for 10 to 20 minutes, embryos were immersed in (1/4 water, 1/4 KOH 10M, 1/2 NaClO 15%) for 30 seconds to facilitate removal of the eggshell (optional), centrifuged, and rinsed in PBST. Prior to each change of solution or rinse, samples were centrifuged for 1 min at 4,200 rpm. This procedure yielded hundreds of embryos per female, and allowed staining of at least half of them. As an alternative procedure we used the freeze crack techniques that work with *C. elegans* embryos but these gave unsatisfactory results, likely due to the smaller size of the *Brugia* embryos. For protocols used to determine the identity of specific embryonic blastomeres and conditions for primary and secondary antibody incubations see Methods S1.

### Live Analysis

For live fluorescent analysis of *Wolbachia* and host nuclei, adult worms were incubated in RPMI medium with 1/10,000 Syto11 (Invitrogen) or vital Hoechst for 30 minutes, and observed as for *C. elegans* on an 2% agarose pad with Sodium Azide 25mM between slide and coverslip (http://www.wormbook.org/chapters/www_intromethodscellbiology/intromethodscellbiology.html). To observe the Secretory-Excretory canal, we added 50µL of Resorufin (Sigma) at 10 µg/mL, 1/10,000 Syto11 for approximately 30 minutes to an hour, and washed the worms in RPMI for 15 minutes. Worms were mounted in PBS and anesthetized with Sodium Azide.

### Microscopy

Confocal microscope images were captured on an inverted photoscope (DMIRB; Leitz) equipped with a laser confocal imaging system (TCS SP2; Leica) using an HCX PL APO 1.4 NA 63 oil objective (Leica) at room temperature. Images in epifluorescence were captured on a Leica DMI 6000B microscope and a Zeiss Axioscope 2 plus microscope.

## Results

### Fertilization and Embryogenesis of *B. malayi*

The fertilization and embryogenesis of *B. malayi* resemble that of other secernentean nematodes. The sperm entry activates the oocyte to complete meiosis I and II and defines the posterior pole of the egg [26], [27]. All examined species of secernentean nematodes undergo asymmetric cleavage leading to early separation of soma and germ line, and establishment of five somatic cell lineages [28]–. This is followed by a developmental phase during which organ identity is specified. Subsequent morphogenetic events such as ventral closure and an elongation phase due to contraction of circumferential actin bundles in the hypodermis lead to newly hatched larvae appearing very similar among nematodes species. It has been shown that despite this high similarity in the anatomy of the first stage larvae (most often the species variation being acquired during larval life), variations can exist from the first asymmetric divisions [31], [32].

**Figure 1:**
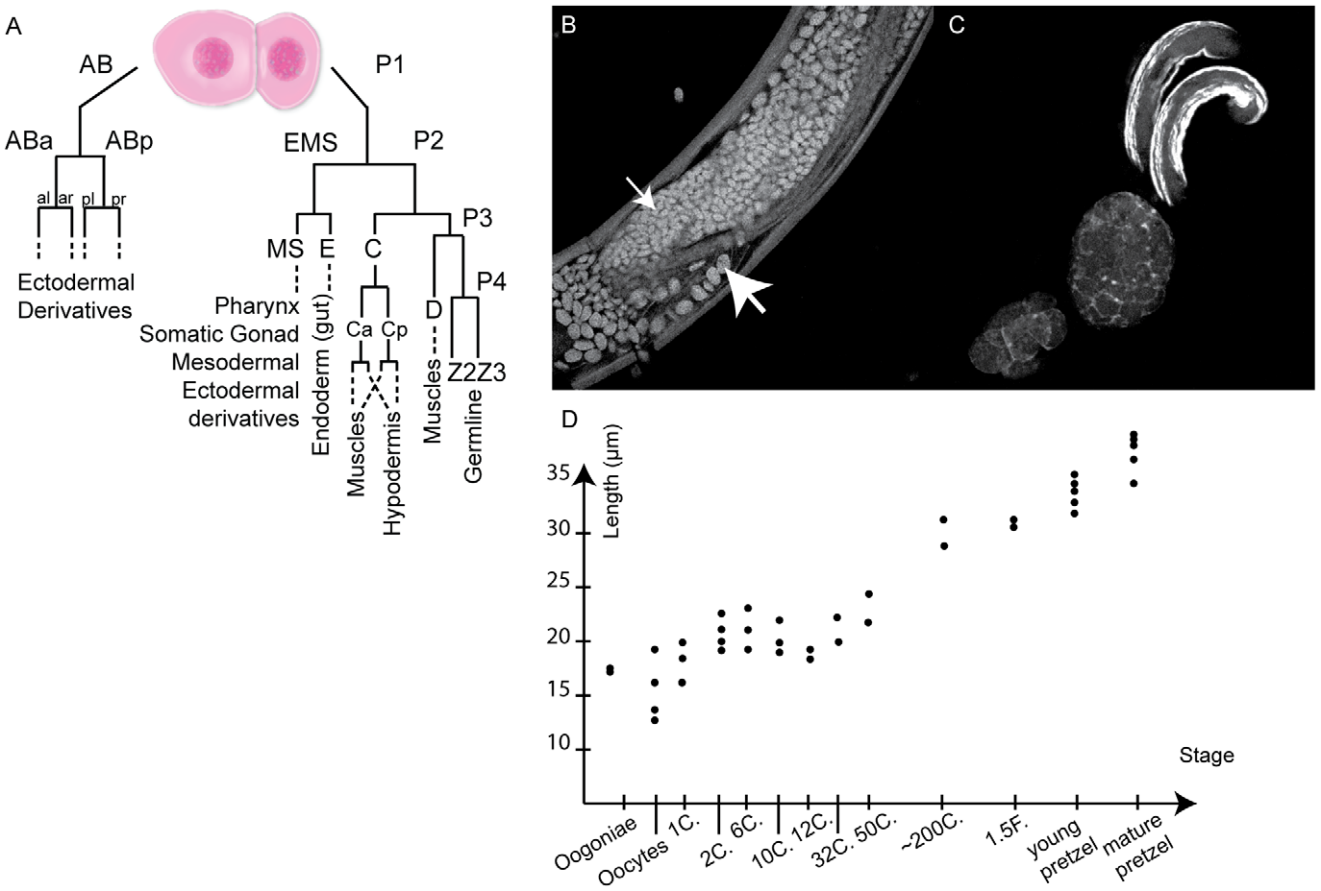
Schematic representation of the first embryonic cleavages in secernentean nematodes, and growth of *Brugia malayi* embryos in utero. (A) Cell linage adapted from [31], [33]. Anterior daughters are to the left, posterior ones to the right. The main derivatives are indicated by dotted lines. (B) Uterus filled with early cleavage embryos. Note the variety of embryo sizes (small and large arrows). (C) Three developmental stages in one merge of confocal stacks (lower left to upper right): embryos at approximately the 12-cell, 100-cell stage prior to morphogenesis, and mature pretzel stage prior to hatching. (D) Length of the *B. malayi* embryo eggshell according to the embryonic stage. In each case, the longest axis of the eggshell was measured. Each data point represents one measurement.

Although the cell lineage of *B. malayi* (a Rhabditia Spirurida of clade III of the Secernentea) has not been established, parallels with the completely defined lineage of *C. elegans* (a Rhabditia Rhabditida of clade V of the Secernentea) are likely [32], [33]. In secernentean nematodes, the first division cleaves the zygote asymmetrically into somatic cell AB and a smaller P1 germ line precursor cell (Fig. 1A). Most of the embryonic ectodermal cells (hypodermal and neuronal cells) are derived from the anterior AB blastomere. The posterior P1 blastomere, after three rounds of division, primarily gives rise to the somatic gonad, pharynx, ectodermal and mesodermal derivatives (MS), gut (E), posterior hypodermal derivatives (C), body wall muscles (D) and P4 blastomeres. During gastrulation, the posterior P4 cell follows the gut precursors inward and divides to produce the two germline precursors Z2 and Z3. Based on the similarity between the *C. elegans* lineage and embryonic maps, putative germ line precursor cells can also be localized (the counterparts of the *C. elegans* Z2 and Z3) during the process of elongation (i.e. when the embryonic tail reaches half the length of the worm body).

In contrast to *C. elegans* embryonic development, *B. malayi* embryos grow and increase their volume in the uterus (Fig. 1B, C). The length of the one-cell egg increases from about 16 µm to 38 µm for an egg containing a mature worm-shaped embryo (Fig. 1D). Thus, unlike *C. elegans*, the *B. malayi* eggshell grows and suggests that uptake of nutrients through the eggshell occurs while the embryo is still in the uterus. These observations may reflect fundamental metabolic differences during embryonic development between the parasitic *B. malayi* and free living *C. elegans*.

### *Wolbachia* Segregation during Fertilization and the Initial Zygotic Divisions

**Figure 2:**
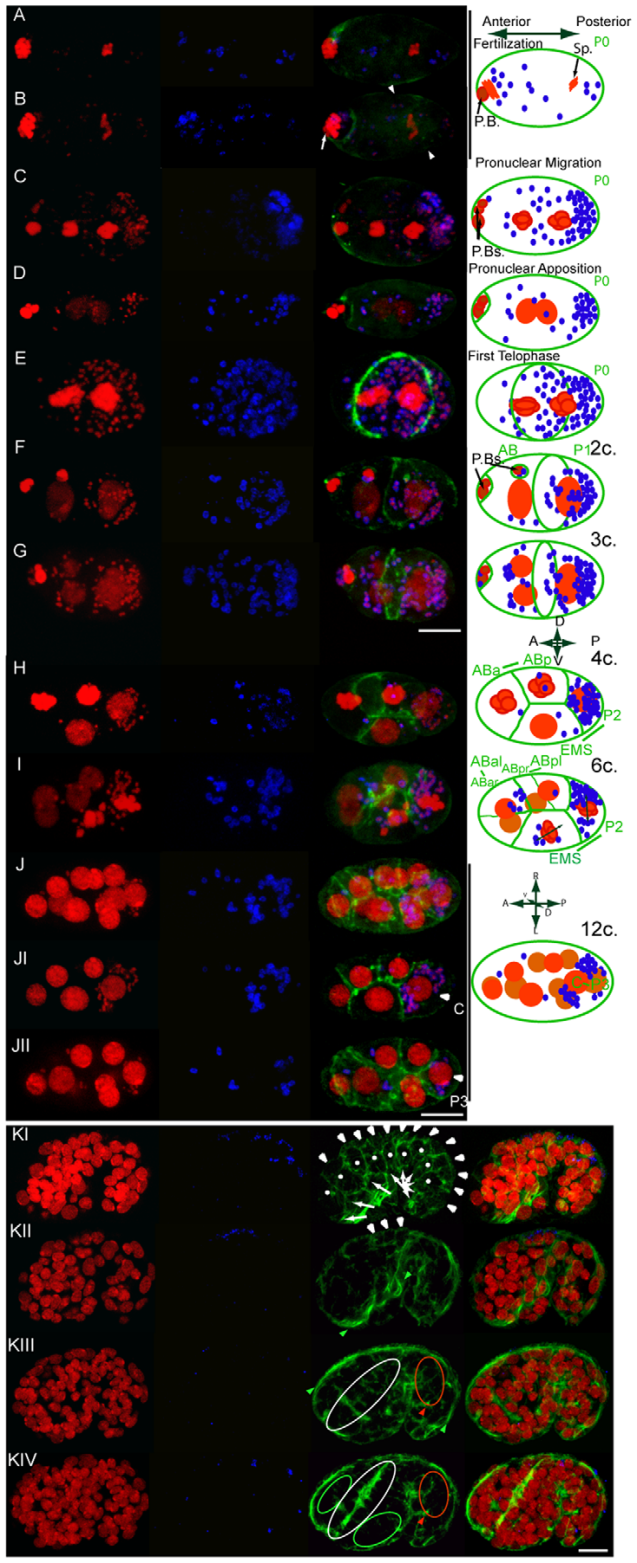
*Wolbachia* concentrate to the posterior pole of the egg and preferentially segregate to the P1 blastomere, to later enrich the C blastomere and subsequently colonize posterior hypodermal cells. Merges of confocal stacks of *B. malayi* eggs and embryos stained for DNA (propidium iodide, red), *Wolbachia* (anti-WSP, blue), and cortical actin (green). (A), completion of meiosis, metaphase I, and (B) completion of metaphase, meiosis II as revealed by the presence of a polar body (PB, white arrow). White arrowheads indicate the pseudocleavage. Maternal chromatin to the anterior pole (left) and paternal chromatin to the posterior (Sp., sperm). (C) Pronuclei migration and (D) Pronuclei apposition while the three polar bodies (PBs) are extruded at the anterior pole. Note that the PBs usually lost at the 4 or 6-cell stage, can detach earlier, probably due to the fixation technique (i.e. (E)). (F) Division of P0 into the anterior AB and the posterior P1 blastomeres. (G) 3-cell stage, AB divides prior P1. (H) 4-cell stage, (I) 6-cell stage, and a (J) 12-cell stage embryo. Partial projections highlighting the dorsal (JI) and ventral (JII) blastomeres. Solid lines connect sister blastomeres on the corresponding schematic drawings. (KI to KIV) Merges of confocal stacks of a comma-stage embryo, from the surface to half depth, anterior to the left. White arrowheads and arrows highlight dorsal and ventral hypodermal cells respectively, and dots indicate lateral hypodermis. Green arrowheads highlight ventral (KII) and dorsal (KIII) muscles. Green, white, and red ovals encompass the neuroblasts, the pharynx, and the gut primordium respectively. The red arrowhead indicates the rectum (KIII, KIV). Scale bar = 6 µm.

We used propidium iodide (PI) to stain the host chromatin and the bacterial DNA, and used an anti-WSP (*wBm* Surface Protein) specific to *Wolbachia* to perform a fluorescent analysis of the *Wolbachia* segregation in the *Brugia* embryo. The anti-WSP revealed that the punctate staining obtained with PI corresponds to the *Wolbachia* DNA and does not stain mitochondrial DNA (i.e. Fig. 2). During fertilization, *Wolbachia* appear distributed throughout the oocyte completing meiosis, although more concentrated in the vicinity of the maternal chromatin in the anterior pole (Fig. 2A, B). This may reflect an interaction with the microtubule spindle as observed at earlier stages (i.e. Fig. S1). As early as the pronuclei migration stage, *Wolbachia* dramatically relocalize towards the posterior pole of the egg (P0, Fig. 2 C to E, n>50).

We then followed *Wolbachia* segregation patterns in the two rounds of division following pronuclear fusion to create a diploid P0 zygotic nucleus. P0 division produces anterior -identified by the localization of polar bodies at the anterior surface (Fig. 1A and Fig. 2F)- and posterior localized, AB and P1 blastomeres respectively. *Wolbachia* always asymmetrically localize in P1 (Fig. 2E, F, n>50). P1 divides to produce EMS and P2 daughter blastomeres (Fig. 1A). *Wolbachia* preferentially segregate to the posteriorly localized P2 blastomere. P2 divides to produce a dorsal C blastomere (Fig. 2JI) and a posterior P3 blastomere (Fig. 2JII). Most of the *Wolbachia* segregate to the C blastomere and a minority segregate to the P3 blastomere.

Although during the first zygotic division, the majority of *Wolbachia* preferentially localize in the P1 blastomere, a few localize to the AB blastomere. Division of the AB blastomere produces daughter blastomeres ABa and ABp (Fig. 2G). *Wolbachia* titer in these descendants is variable but always lower than in the direct descendants of the P1 lineage (Fig. 2H, I).

### *Wolbachia* Segregation during Embryogenesis

In the 12-cell embryo (Fig. 2J) P2 has divided to give dorsally C (Fig. 2JI) and the posterior P3 (Fig. 2JII). Most of the *Wolbachia* are in C, followed by P3. The titer in the AB descendants, MS or E, although variable, is always lower than that in C and P3. In the next rounds of divisions, C divides asymmetrically to give muscle cells and hypodermal cells (Fig. 1A) [33]. However without specific lineage markers, following the descendants of specific blastomeres is not possible after the 12-cell stage. Fortunately morphogenesis, as revealed by phalloidin-based actin staining, in the early *B. malayi* embryo is strikingly similar to that of *C. elegans* (Fig. 2KI to KIV) [34]. At this stage, the cellular proliferation is over, and circumferential actin bundles in hypodermal cells contract, transforming the round-shaped embryo into a worm. In both *B. malayi* and *C. elegans*, the hypodermis is composed of intercalated dorsal cells, lateral and ventral cells organized like the *C. elegans* hyp7, seam and P cells (Fig. 2KI). Under the dorsal and ventral hypodermal cells run the muscle quadrants from the anterior to the posterior (Fig. 2KII,III). Deeper in the embryo, the embryonic neuroblasts, pharyngeal and gut cells can also be localized (Fig. 2KIII, IV). Assuming that the lineage of *B. malayi* is very similar to the established lineage of *C. elegans*, we could verify the vertical transmission of *Wolbachia* in the embryonic blastomeres. Our observations of early stages showed that the *Wolbachia* were diluted out in the AB descendants, and in the MS and E blastomeres (Figs. 1A, 2G to JII). During morphogenesis, we constantly found the *Wolbachia* enriched in the dorsal posterior hypodermis, and absent from nearly all anterior cells including neuroblast and pharyngeal cells, as well as muscle and gut cells (Fig. 2KI to KIV, n>30). This also implies that other asymmetrical segregations of *Wolbachia* occur in the C lineage to exclude them from the C-derived muscle cells and concentrate them in the hypodermis.

**Figure 3:**
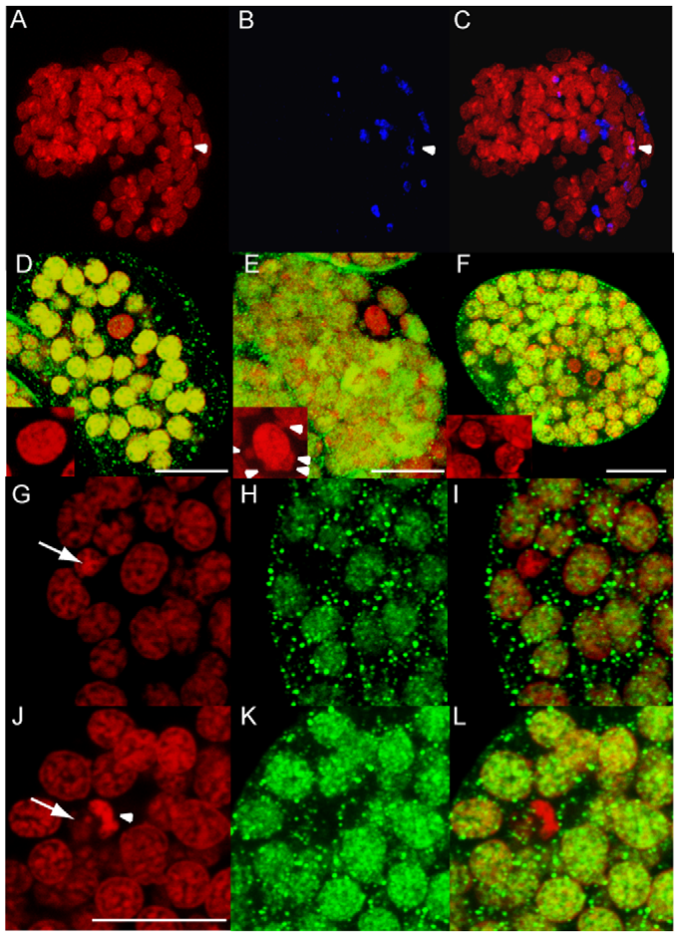
*Wolbachia* are not always detected in the germline precursors. (A) to (C) Merges of confocal stacks of a 1,5 fold stage embryo (when the tail reaches half of the length of the embryo) stained for DNA (propidium iodide, red) and *Wolbachia* (anti-WSP, blue). Infected putative primordial germ cells Z2/Z3 are indicated by an arrowhead. (D) to (L) Merges of confocal stacks of *B. malayi* embryos in gastrulation stained for DNA (PI, red) and with anti-H3K4me2 (green) at the level of the putative P4 blastomere (D,E), or putative Z2 and Z3 (F). (D) and (E) are the earliest stages where the H3meK4-negative P4 blastomere was detected. (G to L) are confocal stacks in cross sections focusing on Z2 or Z3. (D) Non-infected P4; (E) Infected P4 (arrowheads in inset point to *Wolbachia*); (G) Non-infected Z2/Z3; (G to I) Non-infected Z2 or Z3 cell (arrow); (J to L) A *Wolbachia*-infected Z2 or Z3 cell (the arrow points to the cell nucleus and the arrowhead to a cluster of bacteria. Scale bar = 10 µm.

Because the germline precursor P4 has already divided into Z2 and Z3 during morphogenesis and because these cells are often difficult to identify (Fig. 3A to C) we used the anti-histone H3K4me2. It has been demonstrated that in *C. elegans* as well as in *Drosophila melanogaster*, a subset of nucleosome modifications (dimethylation on lysine 4 of histone H3 and acetylation on lysine 8 of histone H4) are absent from germline precursors but present in all the other blastomeres. In *C. elegans* embryos, H3K4me2 marks all the blastomeres, including P4, until it divides symmetrically into Z2 and Z3 [35]. Lysine 4 dimethylation on histone H3 is involved in transcription regulation and its absence reveals transcriptional quiescence [36]. In *B. malayi* however, we found embryos containing only one H3meK4-negative cell, suggesting that the putative P4 blastomere reorganizes its chromatin architecture to enter transcriptional quiescence prior to division (Fig. 3D,E). We found half *Wolbachia*-infected and half non-infected putative P4 blastomeres or putative Z2/Z3 germline cells (Fig. 3D to L). It is likely the embryos with uninfected blastomeres are males and those with infected blastomeres are females.

We also found the average number of *Wolbachia* did not differ from early to mid embryogenesis (70+/−12 (n = 10)) and is in general agreement with the average number detected in microfilariae using qPCR [24]. Although this may suggest that asymmetric relocalization prior to division is the major cause for specific enrichment of a given blastomere, it does not rule out a possible stimulation/repression of bacterial replication due to asymmetrically localized cues. In fact, we noticed that in P2, prior to division, *Wolbachia* appeared as doublets in the enriched antero/dorsal pole while as individual units in the posterior/ventral pole. This was supported by a WSP staining surrounding the doublets, suggesting active replication (Fig. S2A,B).

### Overview of the Anatomy of Adult Female and Male Worms

*B. malayi* adults, like any secernentean nematodes, have a simple and conserved anatomy. Non segmented, these worms have body walls organized in four longitudinal rows of hypodermal chords secreting the cuticle, and separated by four muscle quadrants. Lateral chords contain the excretory-secretory canal, while dorsal and ventral chords surround the nerves. They lack circulatory and respiratory systems. A nerve ring located around the pharynx constitutes the central nervous system (Fig. S3D, E). The triradiate pharynx is connected to the gut. Females have two gonads starting in the posterior and ending in the anterior vulva, while the male has one gonad starting in the anterior and ending in the posterior cloaca.

**Figure 4:**
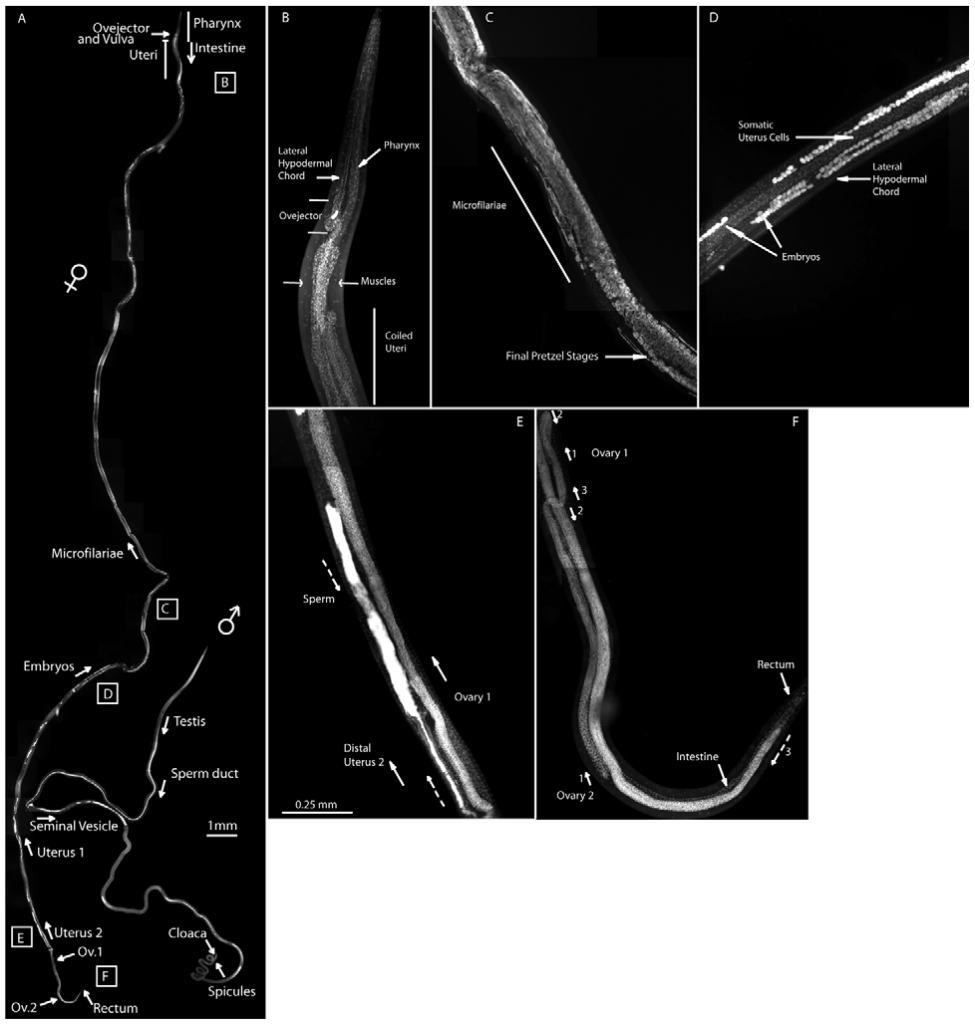
*Brugia malayi* adult anatomy. (A) Overview of the adult anatomy of fixed and DAPI-stained *Brugia malayi* female and male. (B to F) Anatomical details of the *Brugia malayi* female oriented anterior top to posterior bottom. (B) Higher magnification of anterior female reproductive apparatus. A zone of dense somatic nuclei is surrounded by muscles, which have fewer nuclei. (C) Zone of hatching in one of the two uteri. Fully developed pretzel-stage larvae still in their eggshell are at the bottom. Hatched microfilariae that spontaneously align along the antero-posterior are at the top. (D) Developing embryos in the uterus. The perfectly aligned hypodermal nuclei of a lateral chord are visible. (E) Distal-most part of the uterus where sperm has concentrated, brightly stained with DAPI (i.e. in between dotted arrows, also in bright patches in (A) between D and E). (F) Posterior part of the female, showing the two ovaries running back and forth (“ovary 1”, arrows indicating the 180° turns -1, 2 and 3-, idem for “ovary 2”). The amount of sperm, embryos and microfilariae are variable among females.

To determine *Wolbachia* distribution in adult tissues, we stained whole-mount fixed adults either with DAPI or live specimens with the vital dye Syto11 ([37], cf. Fig. 4 and Fig. S4). Detailed measurements of body and tissues features have already been reported [15].

The two female distal gonad arms located in the posterior (several millimeters separate the two ovaries distal ends) coil along an anterior-posterior axis (Fig. 4F). The ovaries lead anteriorly to the two uteri that are also coiled around one another (i.e. Fig. 4B), and filled with sperm that has migrated in their distal parts (Fig. 4A, E). The amount of sperm is variable between females and may reflect the time of observation after copulation. Oogenesis begins at the distal region of the ovaries and as the oocytes mature they are pushed proximally and are fertilized in the distal part of the uteri, where developing embryos are present in the proximal regions (Fig. 4A, C, D; Fig. S5). Thousands of microfilariae are released in the lymph of the host through the ovejector that ends the vulva where the uteri meet, in the anterior part of the female, specifically at the level of the posterior pharynx (Fig. 4A, B, C; Fig. S3A to C). The male gonad consists of a testis posterior to the pharynx of the nematode, connected to the sperm duct which in turn leads to a widened seminal vesicle where mature sperm is stored (Fig. 4A; Fig. S4). The gonad ends in the cloaca where two specialized spicules are used for mating (Fig. S4).

The intestine is a thin empty tube connected at the anterior to the pharynx, and at the posterior to the ventral rectum close to the posterior tip in both male and female worms (Fig. 4A, F; Fig. S6). Gonads and intestine fill the pseudocoel contained within the body wall.

Lateral chords are prominent in *Brugia*. They are formed through fusion of hypodermal cells producing a syncytial chord surrounding the secretory-excretory canal and in between muscle quadrants [32]. The lateral chords project a thin layer of cytoplasm over the muscles to connect the dorsal and ventral chords. These dorsal and ventral chords, containing the dorsal and ventral nerves, are very thin and difficult to observe by differential contrast microscopy but can be revealed by staining the surrounding muscles (Fig. S3F to H). Uteri appear closely apposed or embedded in the lateral chords (Fig. S7). The body wall possesses a slight periodicity, and hypodermal chords and muscle quadrants turn several times around the central axis of the worm between the two tips. The male posterior end is coiled three to four times in the region encompassing the spicules, probably to ensure a better grip during mating (Fig. S4).

### *Wolbachia* in the Somatic Tissues: An Uneven Distribution

**Figure 5:**
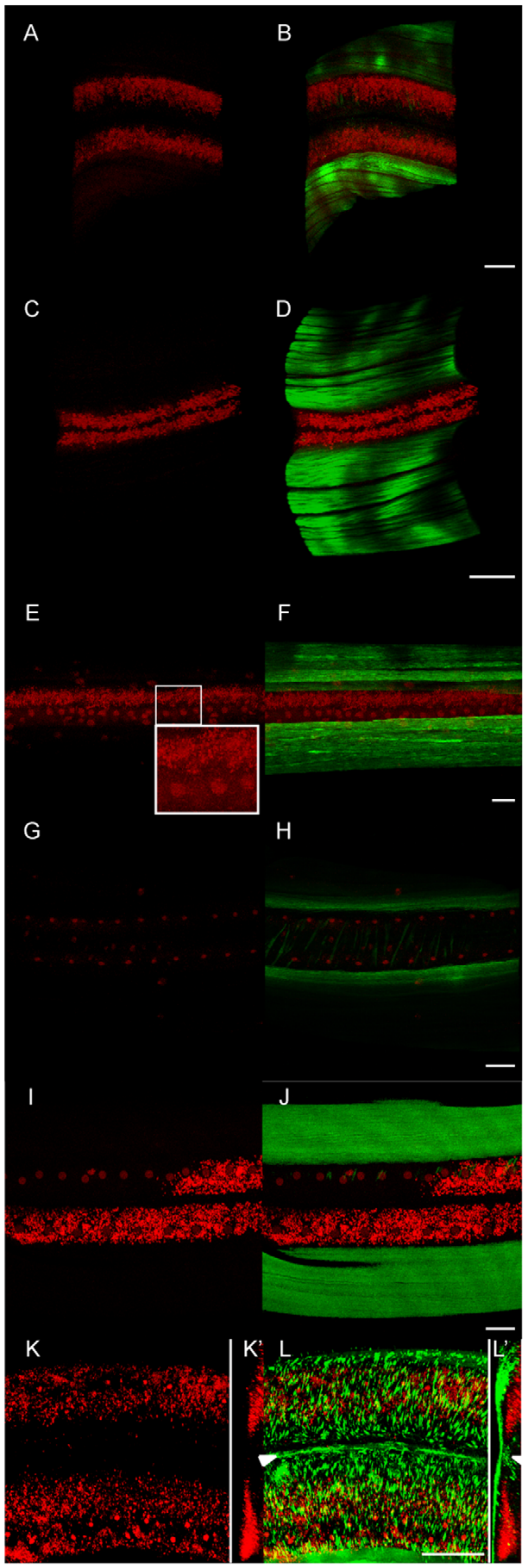
*Wolbachia* concentrate in the hypodermal lateral chords. DNA (propidium iodide, red), and actin (green) stainings of female (A, B) and male (C, D) adult lateral chords. (E, F) The upper but not the lower chord is infected. (G,H) Both chords are uninfected. (H) The phalloidin staining reveals the gonad contractile sheath cells. (I, J) The upper lateral chord is being invaded by *Wolbachia*, revealed by propidium iodide. The uterus is embedded in the chord. (K, L) Surface view of a chord and (K′, L′) 90° projections showing the circumferentially oriented actin bundles in the apical part, while the *Wolbachia*, surround the two rows of hypodermal nuclei (see Material and Methods for technical details) in the basal part, separated by the secretory-excretory canal (arrowheads). Scale bars = 100 µm.

In both male and female worms (n>30), *Wolbachia* concentrate around the two rows of hypodermal nuclei in lateral chords. While most worms displayed two infected lateral chords (Fig. 5A to D), in about 40% only one of the two chords was infected (the left or the right chord). We also observed worms with half of a chord infected (Fig. 5E, F), sometimes in a mosaic pattern (Fig. 5I, J), possibly reflecting the earlier mosaicism in *Wolbachia* segregation during embryonic development. In the chords, the *Wolbachia* like the nuclei are located in the basal part, while the circumferentially oriented actin bundles are in the apical compartment (Fig. 5K to L′). In addition, no adult was found with both lateral chords completely lacking *Wolbachia*, suggesting that *Wolbachia* localization in these chords may be essential for worm survival. Conversely, the *Wolbachia* are completely absent from the intestinal cells, the somatic gonad (gonadal contractile sheath cells and epithelium, Fig. S6) and the muscle quadrants (Fig. 5). It is hard to clearly draw a conclusion on the presence of *Wolbachia* in the nervous system, due to the low quality of the nerve ring chromatin staining with PI but the bacteria appear either absent or at very low titer in this organ (Sup. Fig. 3D, E).

**Figure 6:**
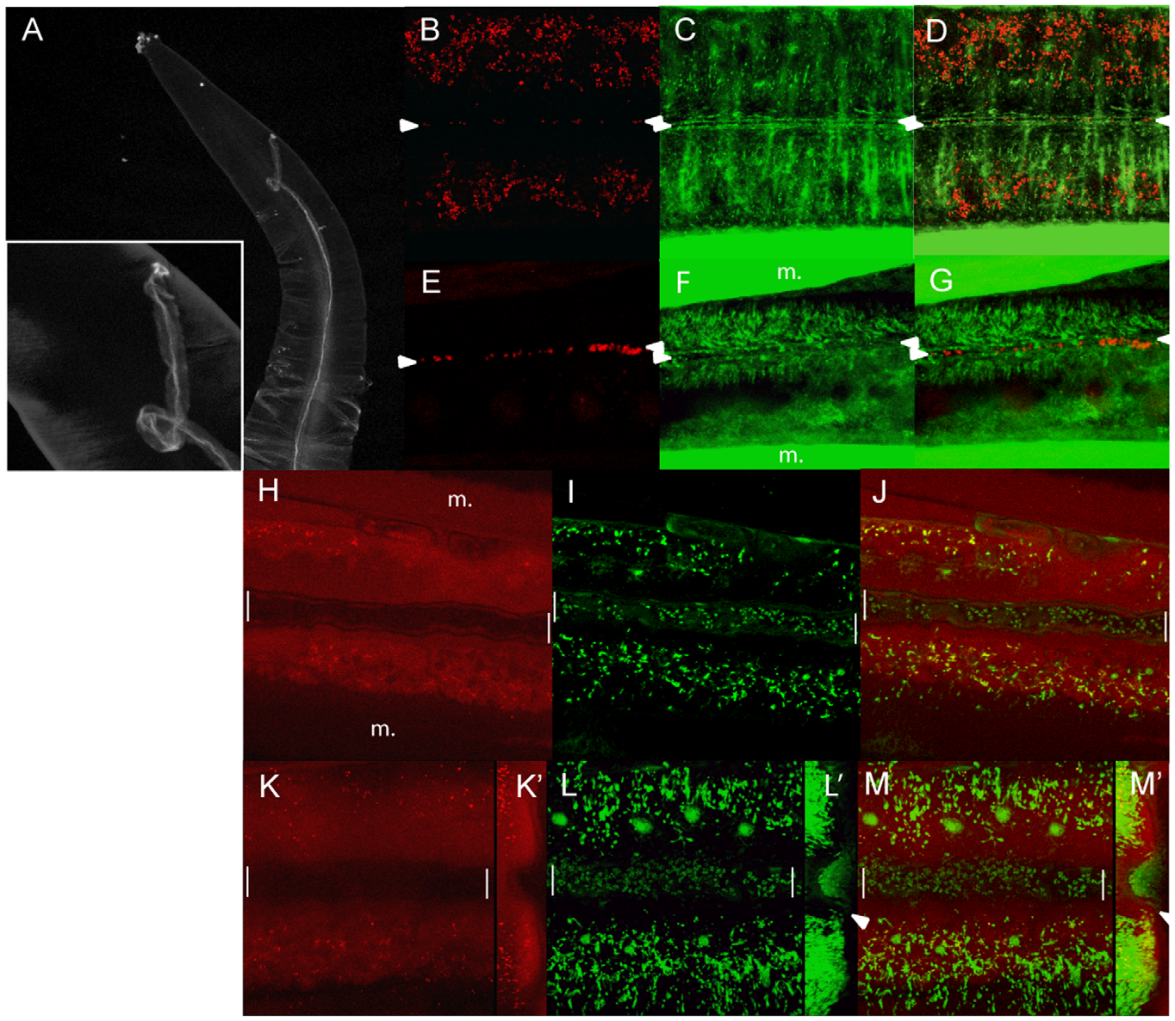
Detection of *Wolbachia* by propidium iodide in the lumen of the secretory-excretory canal. (A) One canal reaching the pore near the mouth, stained with phalloidin without fixation. (B) to (G) Confocal merges at the level of the lumen in fixed females stained for DNA (propidium iodide, red), and cortical actin (green). (B) to (D) At the level of an infected or (E) to (G) non-infected chords. (E) to (G) are close to the pore. m., muscles, arrowheads point to the lumen of the canal. (H) to (M) Live female incubated with resorufin (red) and syto11(green). (H) to (J) one focal plan in the largest section of the canal, (K) to (M) total projection of the canal, (K′) to (M′) 90° projections showing a possible excretion of *Wolbachia revealed by the propidium iodide* (white arrowheads). Note that the canal appears enlarged, as a consequence of the use of Sodium Azide to immobilize the worm (white bars).

We stained the secretory-excretory canals with phalloidin in non-fixed worms, to avoid the overwhelming signal coming from actin-rich tissues (i.e. muscles). Despite variable results, we have been able to locate the secretory-excretory pore close to the mouth (Fig. 6A). By increasing the phalloidin signal in fixed animals, we could reveal the lumen of the canal (Fig. 6B to G, between arrowheads). We found propidium iodide spots present in the lumen of the canal in variable amounts, similar to those in infected parts of chords (Fig. 6B to D), but also in the lumen of the canal in non-infected parts of chords (i.e. Fig. 6E to G). We confirmed these observations on worms kept alive, by visualizing the secretory-excretory canal with the fluorescent marker resorufin. This marker is a substrate of P-glycoproteins and multidrug resistance associated proteins, localized in the apex of polarized cells, involved in excretory processes (cf. [38]). Resorufin concentrates in the chords and neighboring tissues before its excretion via the canal. We combined it with the DNA vital dye syto11 (Fig. 6H to M). Altogether these data suggest that adult *Brugia* may secrete/excrete low numbers of *Wolbachia* into their host.

### *Wolbachia* in the Germline: A Matter of Gender

In the female and male germinal zones, oogoniae or spermatogoniae are partially surrounded by an actin rich membrane connected to the actin-rich central rachis at the distal part of the ovary (Sup. Fig. 5A, C, D). These germ cell nuclei initially organized in a syncitium, then cellularize and detach from the central rachis while migrating proximally towards the uterus or the sperm duct (Fig. S5G to J). All female germ cells are infected and contain an average of 35+/−6.8 *Wolbachia* (n = 13; Fig. S5A to C) in the most distal part of the ovary, before complete cellularization. This suggests a high replication rate of the bacteria in the mitotic region of the ovary (Fig. S5A to C, G, H). More mature oogoniae are located more proximally in the ovary and contain slightly more bacteria (49+/−10, n = 8). Mature oocytes are fertilized when encountering sperm in the distal uterus to give zygotes.

Surprisingly, analogous studies in the male germline revealed no bacteria at any stage of spermatogenesis (n = 3 males). Although it is difficult to distinguish bacteria from mature sperm chromatin, cytological observations at earlier stages of spermatogenesis left no doubt on the absence of *Wolbachia* in the male germline (i.e. Fig. S5D to F, I, J).

## Discussion

### *Wolbachia* Asymmetrically Segregate during Embryogenesis in a Lineage-Specific Manner

**Figure 7:**
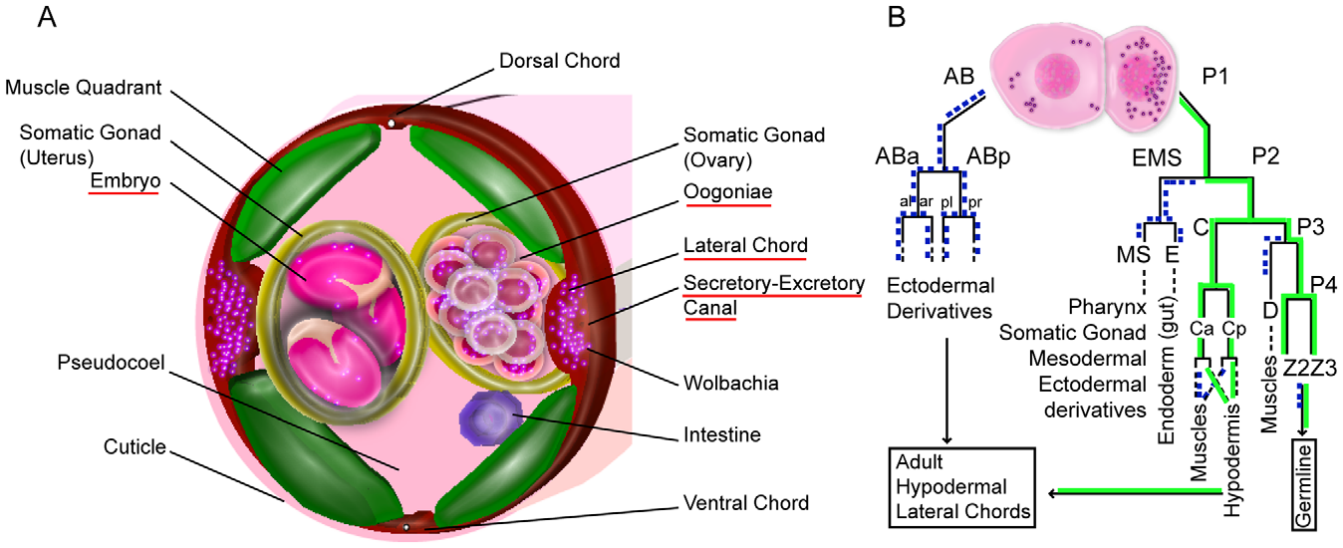
*Wolbachia* localization and segregation patterns followed during early embryogenesis. (A) Schematic drawing of *Wolbachia* localization in a cross section of a *Brugia malayi* adult female. The *Wolbachia* (purple dots) are present in the cell bodies of the hypodermal lateral chords (in red), secretory-excretory canal, in the germline and in embryos. They are absent from the somatic gonad, the muscles and the intestine. (B) Model of likely segregation patterns followed by *Wolbachia* during early embryogenesis to colonize adult tissues. Observation of fixed embryos and adult tissues indicate that *Wolbachia* must follow the germline precursors in females and the C lineage leading to hypodermal cells (green solid lines). In contrast, some blastomere derivatives are not favorable to *Wolbachia* proliferation (i.e. blue dotted lines). In male embryos, the *Wolbachia* may not be maintained in the germline precursors.

Characterization of the transmission mechanisms, distribution pattern and titer of *Wolbachia* in the germline and soma is of primary importance for understanding the biology of the interaction between *Wolbachia* and its host *B. malayi*. We found in *B. malayi* a high *Wolbachia*/ host nuclei ratio in early embryogenesis and in the adult lateral chords. *Wolbachia* were also concentrated in the female germline but absent from the male germline (summarized in Fig. 7A). To understand the origin of this distribution pattern, we examined *Wolbachia* segregation during early embryogenesis. Despite variability in the *Wolbachia* titer among embryos of the same stage, the bacteria were present in all embryonic blastomeres until about the 6-cell stage, but greatly enriched in the posterior pole, following fertilization. Our data indicate that *Wolbachia* are present in the most posterior blastomeres, P2 and EMS at the 4-cell stage, followed by C and P3 in all embryos at the 12-cell stage, that is to say both male and female embryos. Presence in C is the main source of transmission to the hypodermis, while maintenance in P3 and subsequently P4 ensures transmission to the germline (Fig. 7B).

During the mitotic proliferation of oogoniae, association of *Wolbachia* with the mitotic spindle is likely used to ensure an even segregation in the female germline. Interactions of *Wolbachia* with the host microtubules has been well documented in arthropods (i.e. [39]). During fertilization, the primary enrichment could be due to a passive mechanism involving the deep cytoplasmic flow oriented towards the posterior [40]. It is also well established that in *C. elegans* the sperm entry induces partitioning of the evolutionary conserved cell polarizing factors PARs [40], [41]. *Brugia* PAR orthologs and their downstream effectors may be responsible for keeping the *Wolbachia* in the posterior. Asymmetric segregation has been described as a common feature of *Wolbachia* localization in arthropods, such as in *Drosophila* germline and somatic cells, in wasp species and mosquito germlines [42], [43]. Whether the mechanisms used to enrich the posterior of the embryos, to subsequently invade the germ cell precursors, is due to convergent evolution or to common developmental pathways remains to be determined. The latter may provide new targets in anti-*Wolbachia* based therapies in filariasis. An evolutionarily conserved mechanism for posterior localization maybe supported by the mode of invasion of the chords. Instead of invading the AB lineage, the main source of hypodermal blastomeres, *Wolbachia* utilize the posterior C blastomere. It is tempting to speculate that during the evolution of the *Wolbachia*-nematode interaction, the bacteria followed conserved posterior determinants to ensure transmission to the germline, and subsequently acquired an affinity for the C blastomere and its ectodermal derivatives.

In secernentean nematodes, fixed lineages contribute to different types of tissues. This raises the intriguing question of the mechanisms underlying the transmission of the bacteria to the proper differentiated blastomeres. The segregation pattern of *Wolbachia* in the early embryo could result from sensing regulatory networks patterning the embryo, to asymmetrically segregate and proliferate. From the 2 to 12-cell stages, their transmission pattern is very similar to the expression pattern of the *C. elegans* homeodomain protein PAL-1, required for the C-lineage expression [44]. In a second phase, C-derived ectodermal derivatives could trigger *Wolbachia* proliferation [45]. Likewise, germline-specific factors are likely to play a role in segregation in the P germline lineage. It is significant however that some embryos appeared devoid of *Wolbachia* in the P4 blastomere and the Z2/Z3 germline cells, implying a possible loss of *Wolbachia* after establishment of the P3 blastomere and before establishment of Z2/Z3. What happens to these *Wolbachia* remains unclear, they may all segregate into the P4 sister, the D blastomere, to be diluted out without replicating for instance, as observed in the descendants of AB, E or MS. We hypothesize that embryos with and without *Wolbachia* in the P4 blastomere are female and male embryos respectively, since we find a ratio identical to the equal sex ratio described in larvae of the closely related *Brugia pahangi* [46]. Our data do not allow us to rule out possible mechanisms of transcellular invasion from neighboring tissues to non-infected germ cells at later stages, or later loss of *Wolbachia* in the male germline when *Wolbachia* are initially observed in the germline precursors. In *Brugia*, sex determination if of XX/XY type, and males possess a heterogametic pair of chromosomes [47]. *Wolbachia* may sense the gender of the embryo prior to the establishment of the P4 blastomere. Such an early sensing of the embryo's gender may involve interactions with a X- chromosome dosage compensation machinery [48]. In *C. elegans* for instance, this protein complex is active as early as the 30-cell stage, before formation of the P4 blastomere [49], [50].

Based on lessons from *C. elegans* genetics and cell biology on embryonic cell fate establishment, immunofluorescence and RNAi techniques on relevant *Brugia* orthologs should help us to understand the molecular mechanisms of *Wolbachia* transmission.

### Wolbachia Use Cell Fusion to Populate the Hypodermal Chords

We observed the highest bacteria titer in the adult lateral hypodermal chords. Some worms however possess partially infected chords, or one chord lacking *Wolbachia*. This observation may explain the wide range in *Wolbachia* load between individual worms as measured by qPCR [24]. Observations at the embryonic level revealed few infected hypodermal cells, mainly posterior-dorsal, in which the bacteria multiplied (i.e. Fig.2K). A common feature of secernentean nematodes is that during development, hypodermal cells fuse creating a syncytium in the adult [51]. Since we did not find nuclei in the adult ventral and dorsal chords, it is likely that all the hypodermal dorsal and ventral nuclei migrate laterally in *Brugia*. Thus *Wolbachia* may spread through fusion of infected with uninfected hypodermal cells. The developmental timing of hypodermal fusion is unknown. However since we observed *Wolbachia* invasion of lateral chords in young adults (Fig. 5I, J), hypodermal fusion in *Brugia* is likely to have occurred during larval or young adult stages. This would predict a dramatic increase in *Wolbachia* titer per host nuclei during larval stages and early adult, and is supported by quantitative PCR data [25]. Hence, the selective pressure for somatic invasion must be less important than in the germline, since vertical transmission from a single hypodermal cell of a chord is theoretically sufficient to ensure a successful colonization. We have performed our cytological studies in young adults while *B. malayi* can live for many years. This could explain the presence of these partially non-infected chords. It would be interesting to determine whether aged adults contain fully infected lateral chords.

### A Possible Role of *Wolbachia* in the Chords in the Embryonic Development

The nematode hypodermal chords have been shown to play a fundamental function in the metabolism of stored carbohydrate and protein synthesis, as well as the uptake of nutrients via the transcuticular route [51], [52]. *Wolbachia* may participate in these lateral chord functions. Moreover it has been recently demonstrated that part of the stress response induced in *Wolbachia*-depleted *B. malayi* by tetracycline is an upregulation of amino acids synthesis and protein translation, suggesting an initial compensation for the lack of *Wolbachia* [53]. Depletion of *Wolbachia* with antibiotics has been shown to reduce the production of microfilariae and to affect embryogenesis [53]–[55]. This last study also shows that tetracycline treatments result in *Wolbachia* degeneration in the germline and embryos prior to *Wolbachia* loss in the lateral chords. Defects in embryogenesis may still be due to a perturbed metabolism starting at the level of the hypodermal chords rather than a direct effect on the few *Wolbachia* present in embryonic hypodermal and germ line cells. Support for this idea comes from the fact that *Wolbachia* are present exclusively in the female germ line and not in the male germ line. Thus while *Wolbachia* are transmitted vertically through the female germline, they may not be necessary for germline development. Selective pressure in the germline may be greatly reduced in endosymbionts such as *Wolbachia* that are involved in metabolic mutualism. In contrast, *Wolbachia* are parasitic in many arthropod species and accordingly have a profound influence on host germline function [43]. Second, we observed an increase in embryo size during development suggesting nutrient uptake from the uterus. Third, both in live specimens and in whole mount fixed adults a tight association between lateral chords and the uterus was observed, arguing for a role of the chords in supplying the production demands of microfilariae (i.e. Fig. 5H and Fig. S7).

### Adult Worms May Trigger the Host Immune Response by Directly Secreting *Wolbachia*

It has been established that *Wolbachia* release in the human body, presumably from degenerating worms, has a crucial impact on the development of river blindness and lymphatic filariasis, by activating the host immune response [3], [12], [13], [14]. We detected variable amounts of *Wolbachia* in the secretory-excretory canals, present in the chords, even in non-infected regions of the chords. This suggests that in addition to degenerating worms, live adults may release *Wolbachia*, through the excretory pore. PCR analysis of short term *in vitro* culture supernatant was unable to detect *Wolbachia* DNA, although 90 *Wolbachia* proteins were detected in ES products [56]. Furthermore, immunohistochemistry of *O. volvulus* does not detect the abundant release of *Wolbachia* into the surrounding tissues [57]. Nevertheless, low numbers of *Wolbachia* and/or their products may be released via the excretory/secretory canal as previously hypothesized [58], and act as an additional source of immunostimulatory components that contribute to the known innate and adaptive immune responses typical of filarial infections [59]–[61].

## Supporting Information

Figure S1 *Wolbachia* concentrate to the astral and spindle microtubules in oogoniae during mitosis. Merges of confocal stacks of *B. malayi* oogoniae stained for DNA (propidium iodide, red), *Wolbachia* (anti-WSP, blue), and microtubules (green). (A) Oogonia in S phase. (B) Oogonia in metaphase. (C) Oogoniae in anaphase. Arrowheads point to *Wolbachia* associated with the spindle, and arrows highlight *Wolbachia* around the asters. The figures of division and the absence of polar bodies strongly suggest that these oogoniae are in the phase of mitotic proliferation. Note than in (B) and (C) sperm cells are in the top right–present on slide after dissection of the gonads. Scale bars = 5 µm.
(1.99 MB PDF)

Figure S2 Stimulation of *Wolbachia* replication by host factors may participate to the asymmetrical enrichment. (A) Merge of confocal stacks of the P2 blastomere of the 6-cell stage embryo shown in Fig. 2H. (B) One focal plane showing doublets of *Wolbachia* in the antero-dorsal pole (arrows) or single bacteria in the postero-ventral pole (arrowheads) pole of P2.
(0.55 MB PDF)

Figure S3 Anterior histological observations in *Brugia* female. (A to C) Phalloidin (A) and PI stainings (B) of the vulva and the ovejector, in front of the pharynx. (D, E) Phalloidin and PI stainings of the nerve ring (arrows). (F to H) Phalloidin (F) and propidium iodide (G) stainings showing the dorsal chord (arrow). Neither *Wolbachia* nor nuclei are found in the narrow chord.
(3.15 MB PDF)

Figure S4 Anatomical details of the *Brugia malayi* male. Syto11 staining of a male observed by epifluorescence. Anterior is at the bottom right. Enlargements show key stages of spermatogenesis, and the most posterior coiled part.
(2.46 MB PDF)

Figure S5 *Wolbachia* are present in high number in the female germline, but absent from the male germline. (A to C) Distal ovary or (D to F) distal testis stained for DNA (propidium iodide, grey and red channels), actin (green), with the addition of anti-acetylated histone H4 staining on panel (C) and (H) in green to discriminate *Wolbachia* DNA (white arrowheads in (A)) from host chromatin. In (B) the central actin-rich rachis is clearly visible. Higher magnifications in a more proximal localization in a female and male gonad of a cellularized oogonium (G, H) or of cellularized spermatocytes (I, J). In (G) and (H) somatic gonad nuclei in the background are not surrounded by bacteria. Note in (I) and (J) the presence of mature sperm cells brightly stained with propidium iodide (arrows). Scale bars = 12 µm.
(2.68 MB PDF)

Figure S6 *Wolbachia* are absent from the somatic gonads and intestine. Merges of confocal stacks of tissues stained for DNA (propidium iodide, (A) and (D)), actin (green, (B) and (E)), and *Brugia* chromatin (anti-acetylated Histone H4, green, (B) and (E)). (A to C) Outer view of a gonad showing epithelial cells (arrowheads) and contractile sheath cells (arrows). (D to F) Sagital view of intestinal cells in the widest part of the lumen. Scale bars = 10 µm.
(1.00 MB PDF)

Figure S7 Vital Hoechst staining (left panels) and DIC (center) imaging of a lateral hypodermal chord above a uterus at mid length of a female (merge on the right). The depth of focus from the cuticle is indicated on the left panels in µm. (A) The somatic gonad (s.g.) is embedded in the lateral chord (l.c.) and the pseudocoel is not visible between these tissues at this level. Nuclei of the contractile sheath cells are visible (arrows). (B) Nuclei of the lateral chord are aligned in two deep grooves where lie the hypodermal cell bodies (B and C, arrows). *Wolbachia* appear as a granulated staining in the lateral chords (i.e. (A) white circle). (D) A thin projection of hypodermal cytoplasm covers the uterus and contains the secretory-excretory canal (s.e.c, double white arrow) appearing as a dark line on left panels of (D) and (E). In (D) and (E) the hypodermal cytoplasmic projections above the uterus and the muscles do not contain any bacteria, probably because of spatial constraints. (F) The cuticle secreted by the hypodermis. Scale bar = 50 µm.
(0.20 MB PDF)

Methods S1 (0.03 MB DOC)
