# Supplementary figures and images for: Asymmetric Wolbachia Segregation during Early Brugia malayi Embryogenesis Determines Its Distribution in Adult Host Tissues
Source: PLoS Negl Trop Dis. 2010 Jul 27;4(7):e758. doi: 10.1371/journal.pntd.0000758 (PMC2910707; doi:10.1371/journal.pntd.0000758)

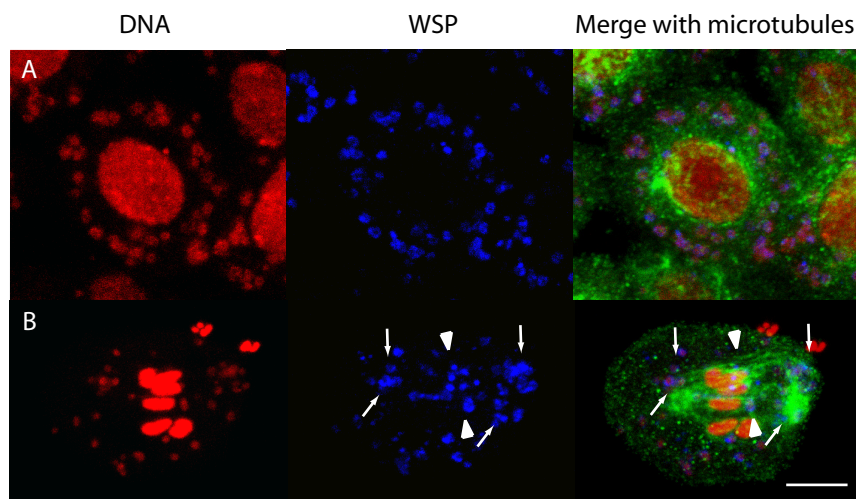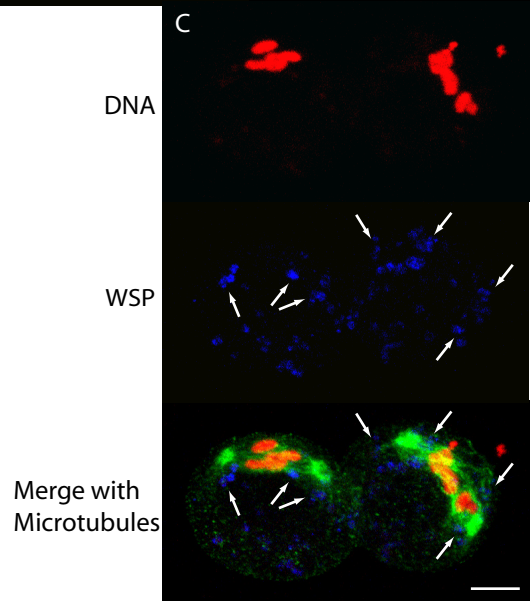

Supplement: Figure S1 — Wolbachia concentrate to the astral and spindle microtubules in oogoniae during mitosis. Merges of confocal stacks of B. malayi oogoniae stained for DNA (propidium iodide, red), Wolbachia (anti-WSP, blue), and microtubules (green). (A) Oogonia in S phase. (B) Oogonia in metaphase. (C) Oogoniae in anaphase. Arrowheads point to Wolbachia associated with the spindle, and arrows highlight Wolbachia around the asters. The figures of division and the absence of polar bodies strongly suggest that these oogoniae are in the phase of mitotic proliferation. Note than in (B) and (C) sperm cells are in the top right–present on slide after dissection of the gonads. Scale bars = 5 µm. (1.99 MB PDF) [file pntd.0000758.s001.pdf]

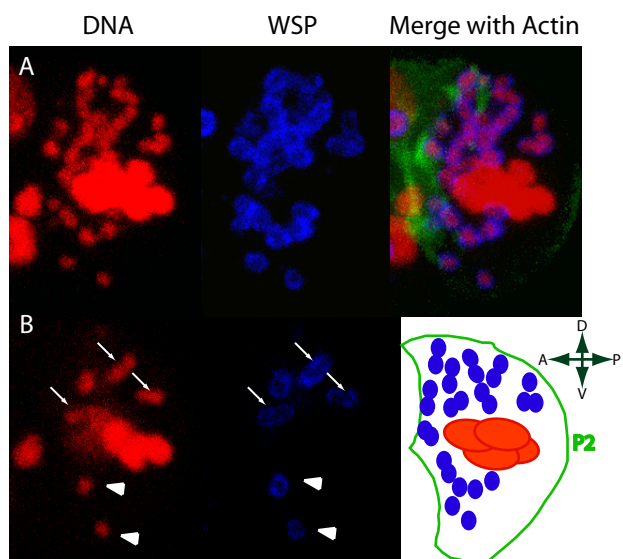

Supplement: Figure S2 — Stimulation of Wolbachia replication by host factors may participate to the asymmetrical enrichment. (A) Merge of confocal stacks of the P2 blastomere of the 6-cell stage embryo shown in Fig. 2H. (B) One focal plane showing doublets of Wolbachia in the antero-dorsal pole (arrows) or single bacteria in the postero-ventral pole (arrowheads) pole of P2. (0.55 MB PDF) [file pntd.0000758.s002.pdf]

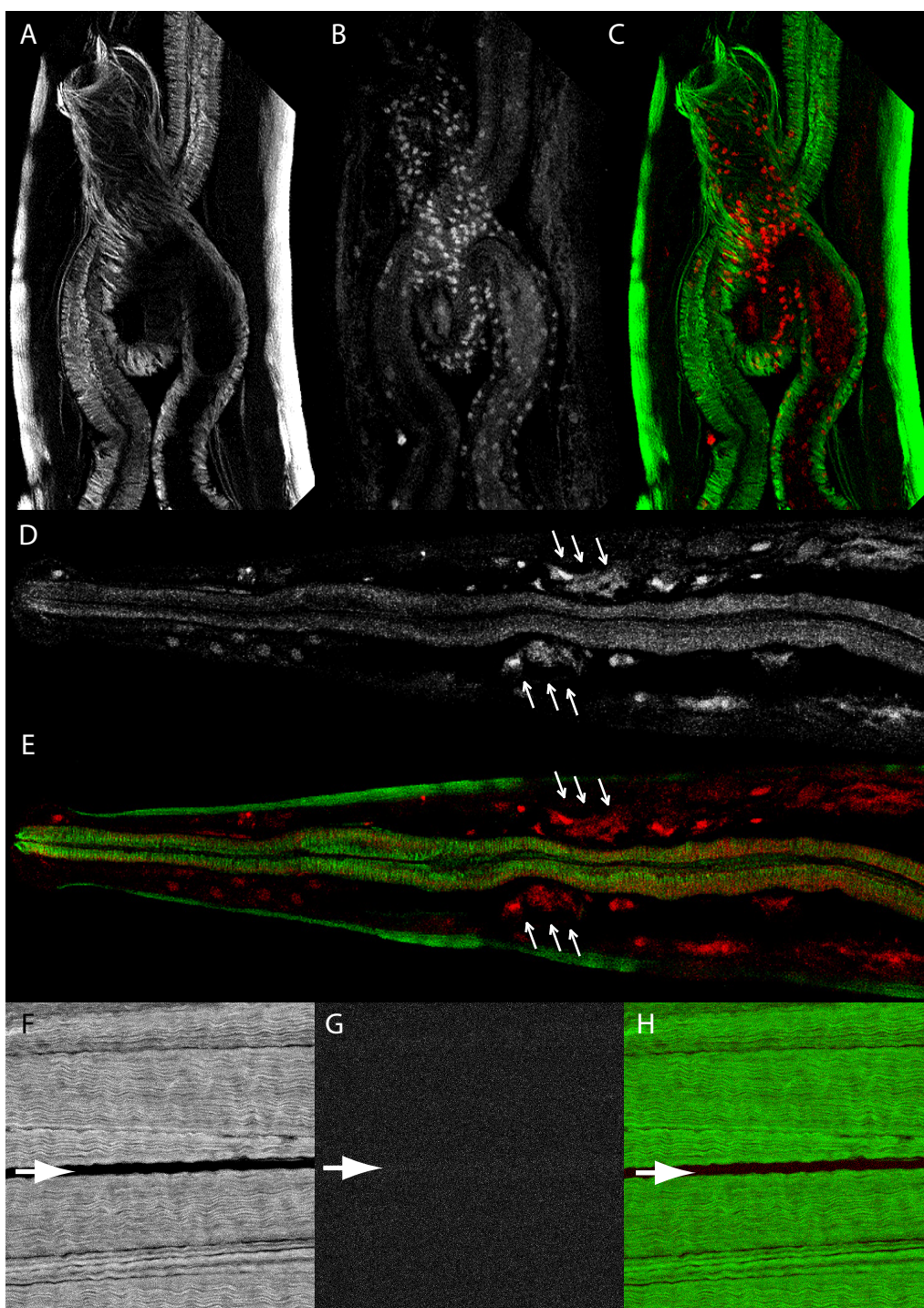

Supplement: Figure S3 — Anterior histological observations in Brugia female. (A to C) Phalloidin (A) and PI stainings (B) of the vulva and the ovejector, in front of the pharynx. (D, E) Phalloidin and PI stainings of the nerve ring (arrows). (F to H) Phalloidin (F) and propidium iodide (G) stainings showing the dorsal chord (arrow). Neither Wolbachia nor nuclei are found in the narrow chord. (3.15 MB PDF) [file pntd.0000758.s003.pdf]

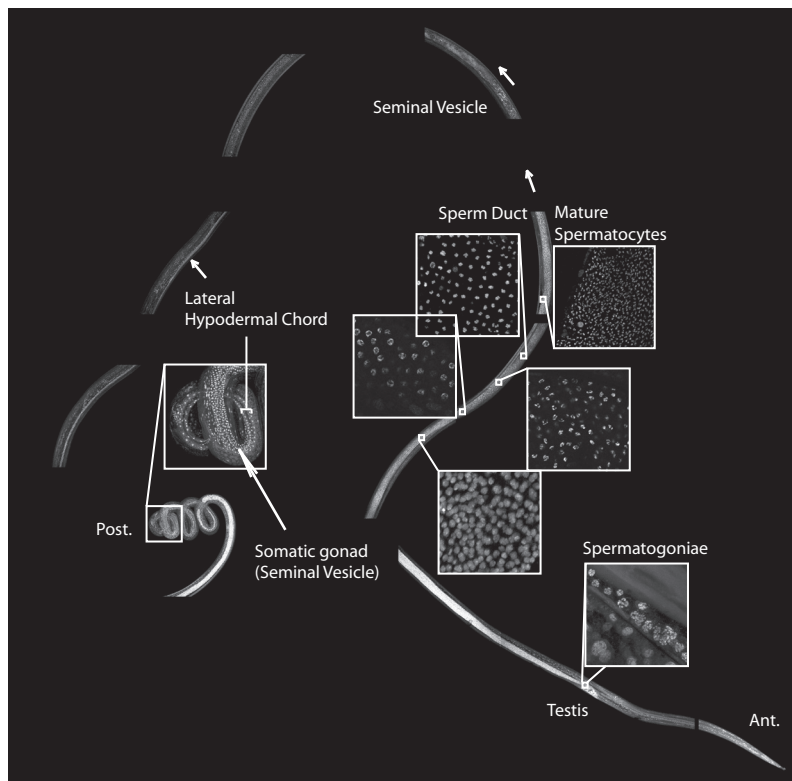

Supplement: Figure S4 — Anatomical details of the Brugia malayi male. Syto11 staining of a male observed by epifluorescence. Anterior is at the bottom right. Enlargements show key stages of spermatogenesis, and the most posterior coiled part. (2.46 MB PDF) [file pntd.0000758.s004.pdf]

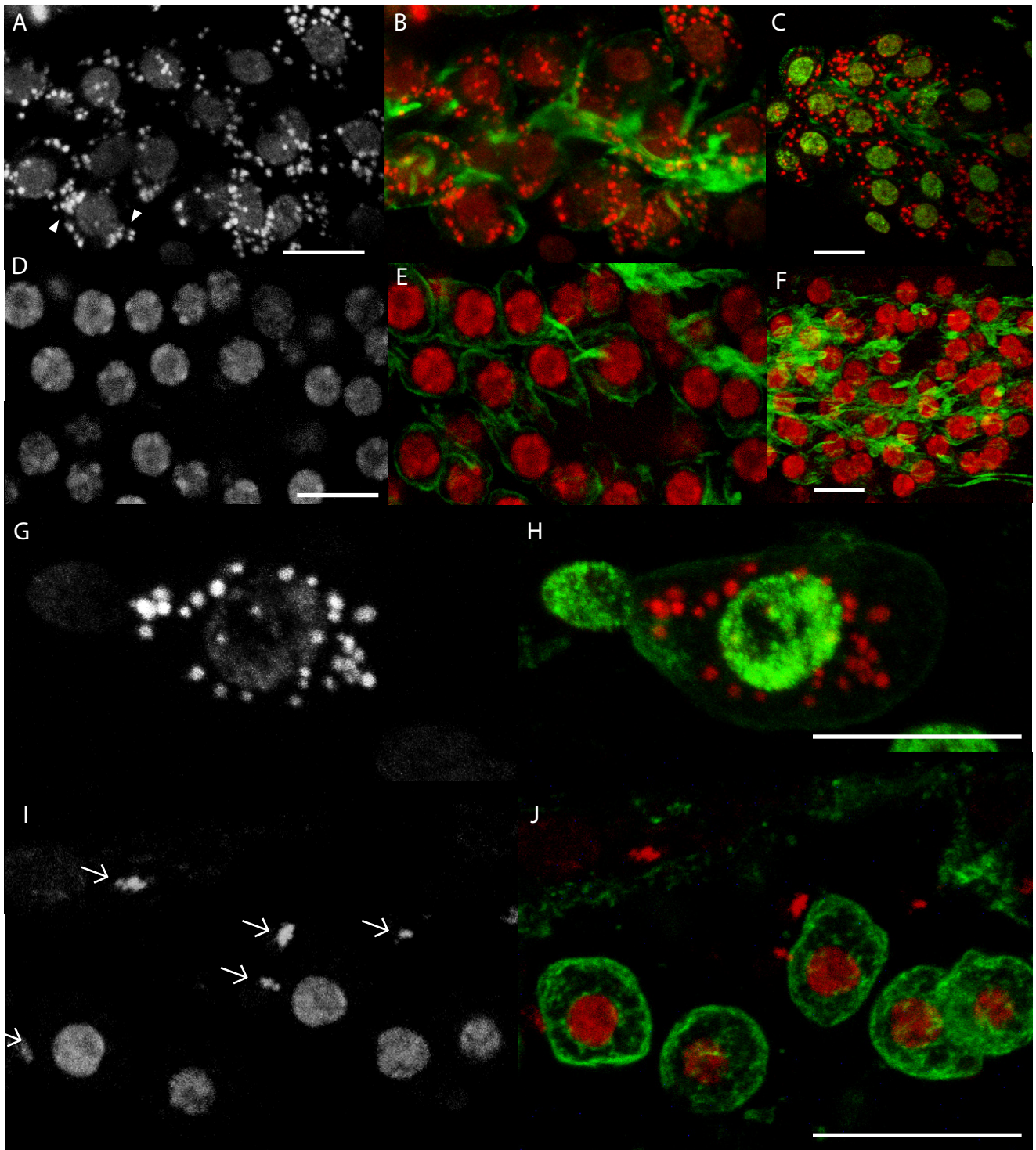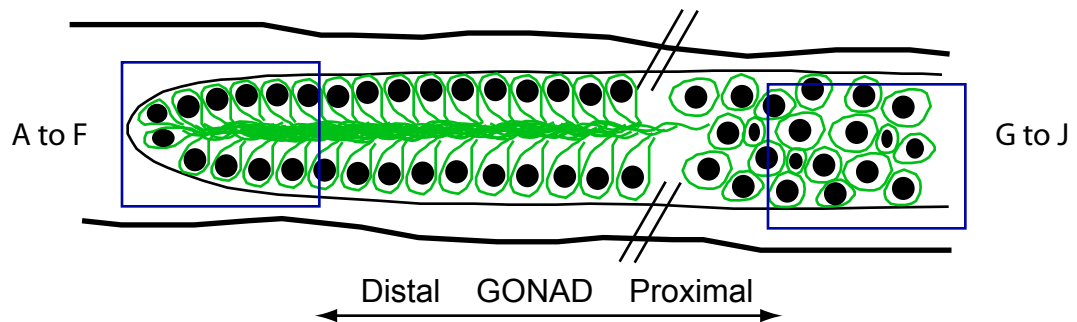

Supplement: Figure S5 — Wolbachia are present in high number in the female germline, but absent from the male germline. (A to C) Distal ovary or (D to F) distal testis stained for DNA (propidium iodide, grey and red channels), actin (green), with the addition of anti-acetylated histone H4 staining on panel (C) and (H) in green to discriminate Wolbachia DNA (white arrowheads in (A)) from host chromatin. In (B) the central actin-rich rachis is clearly visible. Higher magnifications in a more proximal localization in a female and male gonad of a cellularized oogonium (G, H) or of cellularized spermatocytes (I, J). In (G) and (H) somatic gonad nuclei in the background are not surrounded by bacteria. Note in (I) and (J) the presence of mature sperm cells brightly stained with propidium iodide (arrows). Scale bars = 12 µm. (2.68 MB PDF) [file pntd.0000758.s005.pdf]

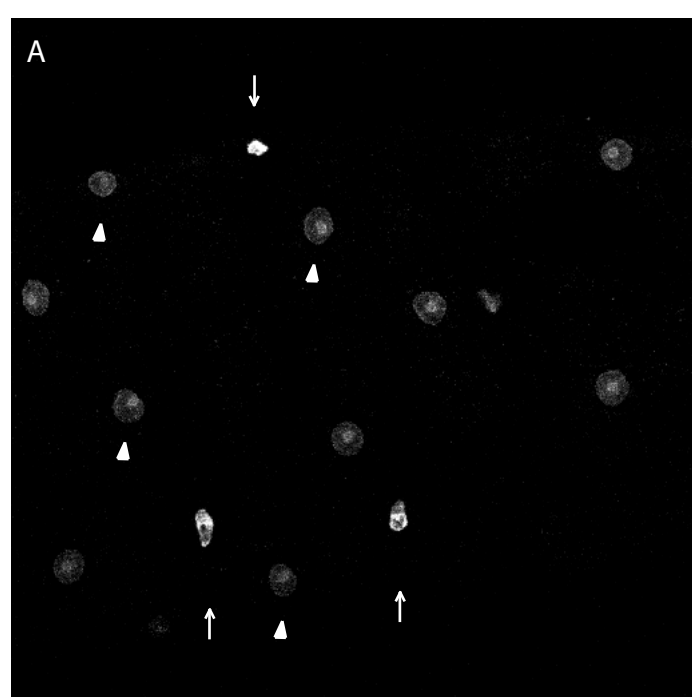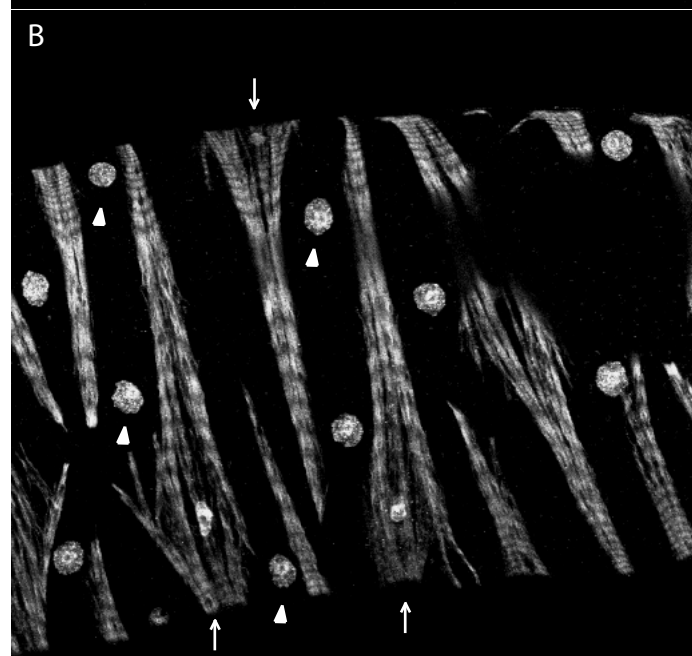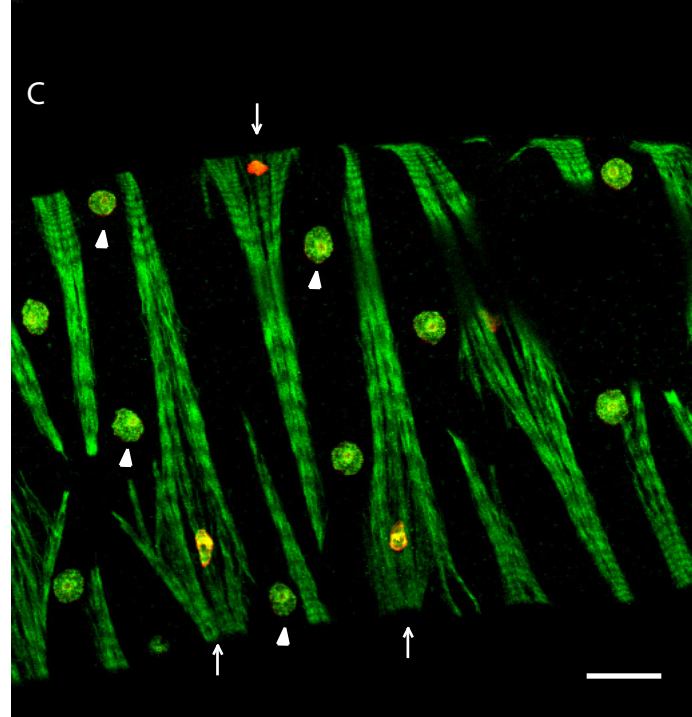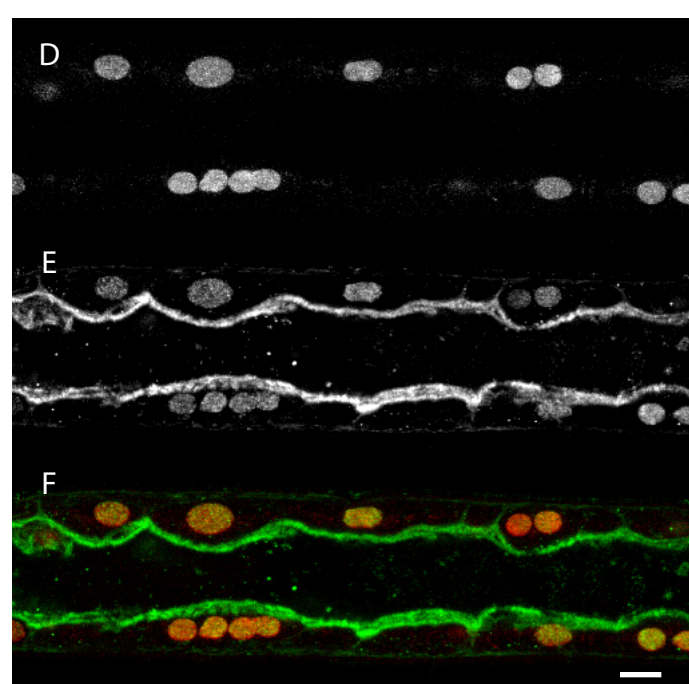

Supplement: Figure S6 — Wolbachia are absent from the somatic gonads and intestine. Merges of confocal stacks of tissues stained for DNA (propidium iodide, (A) and (D)), actin (green, (B) and (E)), and Brugia chromatin (anti-acetylated Histone H4, green, (B) and (E)). (A to C) Outer view of a gonad showing epithelial cells (arrowheads) and contractile sheath cells (arrows). (D to F) Sagital view of intestinal cells in the widest part of the lumen. Scale bars = 10 µm. (1.00 MB PDF) [file pntd.0000758.s006.pdf]

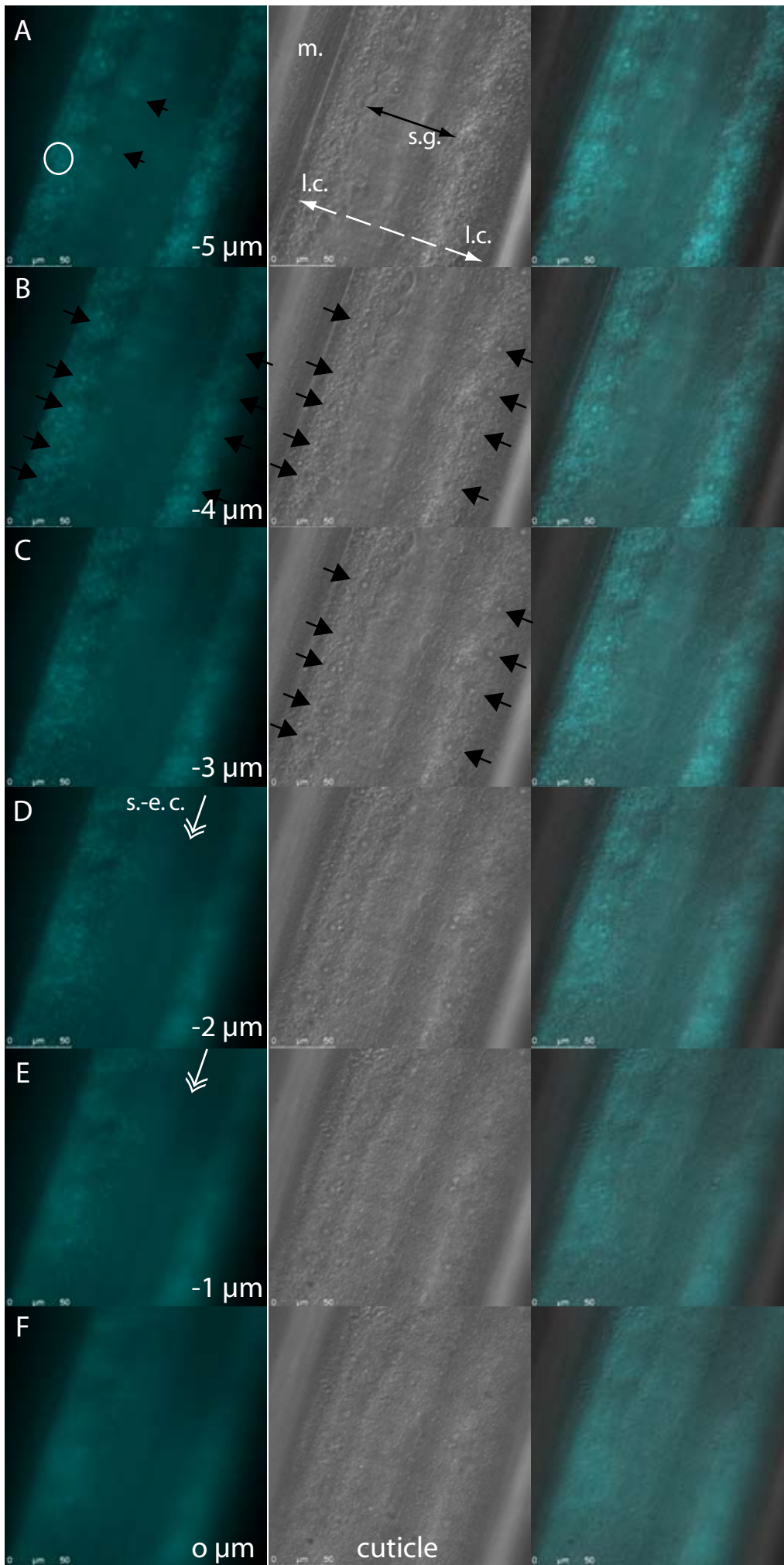

Supplement: Figure S7 — Vital Hoechst staining (left panels) and DIC (center) imaging of a lateral hypodermal chord above a uterus at mid length of a female (merge on the right). The depth of focus from the cuticle is indicated on the left panels in µm. (A) The somatic gonad (s.g.) is embedded in the lateral chord (l.c.) and the pseudocoel is not visible between these tissues at this level. Nuclei of the contractile sheath cells are visible (arrows). (B) Nuclei of the lateral chord are aligned in two deep grooves where lie the hypodermal cell bodies (B and C, arrows). Wolbachia appear as a granulated staining in the lateral chords (i.e. (A) white circle). (D) A thin projection of hypodermal cytoplasm covers the uterus and contains the secretory-excretory canal (s.e.c, double white arrow) appearing as a dark line on left panels of (D) and (E). In (D) and (E) the hypodermal cytoplasmic projections above the uterus and the muscles do not contain any bacteria, probably because of spatial constraints. (F) The cuticle secreted by the hypodermis. Scale bar = 50 µm. (0.20 MB PDF) [file pntd.0000758.s007.pdf]
