# Supplementary material for: Asymmetric Wolbachia Segregation during Early Brugia malayi Embryogenesis Determines Its Distribution in Adult Host Tissues
Source: PLoS Negl Trop Dis. 2010 Jul 27;4(7):e758. doi: 10.1371/journal.pntd.0000758 (PMC2910707; doi:10.1371/journal.pntd.0000758)
Supplement: Methods S1 — (0.03 MB DOC) [file pntd.0000758.s008.doc]

## Methods S1

Antibody incubations:

We stained *B. malayi* embryos with anti-

*C. elegans* monoclonal antibody MH27 (1/500) which recognizes the epithelial cell junction marker AJM-1 (Francis and Waterston, 1991). We also tried polyclonal antibodies directed against C. elegans germ line granule proteins GLH-4 (1/1000) and PGL-1 (1/10,000) (kindly provided by Susan Strome) in order to identify germ cells (Kawasaki et al., 1998; Kuznicki et al., 2000). None of these antibodies showed any cross reactivity with Brugia antigens (data not shown). However rabbit polyclonal antibodies directed against modified histones (anti-H3K4me2 at 1:250 (Davis Allis), polyclonal anti-tetra acetylated H4 (1:300, Upstate) worked well.

A chicken polyclonal antibody directed against the major *Wolbachia* surface protein (anti-WSP 1/100, provided by G. Tzertzinis, NEB) was used for immunofluorescent detection of *Wolbachia.*

Cy5 goat anti-rabbit IgG and Alexa Fluor 488 goat anti–mouse IgG antibodies were used at 1:150 (Invitrogen), Cy5 donkey anti-chicken IgG at 1:200 (Jackson Immunoresearch) for the anti-WSP. DNA was detected with propidium iodide (Molecular Probes, 1.0 mg/mL solution) after a 20-minute incubation in PBS (1:50) and a 5-minute wash. Alexa Fluor 488-conjugated phalloidin was used to stain F-actin. This was added in combination with secondary antibodies. In order to improve the staining of the circumferentially oriented actin bundles in the hypodermis and decrease the robust staining of the surrounding muscle quadrants (i.e. Fig. 9 K to L'), living worms were incubated with Alexa Fluor 488-conjugated phalloidin added to the RPMI culture medium (Sigma) at 37^0^C for 1 hour, prior to direct observation or fixation for additional stainings.

Incubations with primary antibodies were performed overnight at 4^0^C in rotating Eppendorf tubes. followed by 3 washes of 5 minutes each, and incubations with secondary antibodies were performed either at 37^0^C for 3 hours or overnight at 4^0^C. For deep tissue-phalloidin staining, best results were obtained with an overnight incubation at 4^0^C under rotation. Incubations with antibodies and washes were done with PBST.

Buffers:

-M9 buffer (1X): 3g KH2PO4, 6g Na2HPO4, 5g NaCl, 1 ml 1 M MgSO4, H2O to 1 litre. Sterilize by autoclaving.

-PBS (1X): 137 mM NaCl, 2.7 mM KCl, 4.3 mM Na2HPO4, 1.47 mM KH2PO4. Adjust to a final pH of 7.4.

-PBST (1X): PBS + 2% Bovine Serum Albumine (Fraction V, Fisher), 0.1% Triton (Fisher).
